# Patient Activation in Type 2 Diabetes: Does It Differ between Men and Women?

**DOI:** 10.1155/2016/7386532

**Published:** 2016-08-30

**Authors:** Steven H. Hendriks, Laura C. Hartog, Klaas H. Groenier, Angela H. E. M. Maas, Kornelis J. J. van Hateren, Nanne Kleefstra, Henk J. G. Bilo

**Affiliations:** ^1^Diabetes Centre, Isala, 8000 GK Zwolle, Netherlands; ^2^Department of General Practice, University of Groningen and University Medical Center Groningen, 9713 GZ Groningen, Netherlands; ^3^Department of Cardiology, Radboud University Medical Center, 6525 HP Nijmegen, Netherlands; ^4^Langerhans Medical Research Group, 8025 BT Zwolle, Netherlands; ^5^Department of Internal Medicine, University of Groningen and University Medical Center Groningen, 9713 GZ Groningen, Netherlands; ^6^Department of Internal Medicine, Isala, 8025 AB Zwolle, Netherlands

## Abstract

*Background.* Aim was to investigate whether the degree of patient activation of patients with type 2 diabetes (T2D) is different between men and women. Furthermore, we investigated which factors are associated with patient activation in men and women.* Methods.* This cross-sectional study included 1615 patients with T2D from general practices. Patient activation was measured with the Patient Activation Measure (PAM) questionnaire. Multivariate linear regression analyses were used to investigate the association between gender and patient activation. Stratified analyses according to gender were performed to investigate which factors are associated with patient activation.* Results.* No association between gender and PAM score was found after adjustment for all selected confounders (*p* = 0.094). In men, lower age (*p* = 0.001), a higher WHO-5 score (*p* < 0.001), and a lower BMI (*p* = 0.013) were associated with a higher PAM score. In women, a higher WHO-5 score (*p* < 0.017) and the absence of macrovascular complications (*p* < 0.031) were associated with a higher PAM score.* Conclusion.* There is no difference in the degree of patient activation of men and women with T2D. Age, well-being, and BMI were found to be associated with patient activation in men, whereas well-being and macrovascular complications were found to be associated with patient activation in women.

## 1. Introduction

Patient participation is essential to achieve and maintain good overall and diabetes control. The Association of American Diabetes Educators (AADE) has defined 7 self-care behaviours, which are essential for successful and effective diabetes self-management. These are healthy eating, being active, taking medication, monitoring, problem solving, healthy coping, and reducing risks of diabetes-related complications [1].

Not all subjects with type 2 diabetes (T2D) are equally capable of performing these self-care tasks as performing these tasks requires knowledge, discipline, and perseverance. To measure someone's ability to take control of his or her own health, Hibbard et al. developed the Patient Activation Measure (PAM) questionnaire [2, 3]. They have defined patient activation as someone's knowledge, skills, and confidence needed for self-management [2]. According to the developers, patients go through four stages of patient activation and every stage needs a different approach. Patient activation starts with convincing patients that their own actions can have a positive influence on health. Subsequently, attention should be paid to obtaining an adequate knowledge base for making good choices. Thirdly, attention should be given to confidence building by achieving success in very small behavioural modification steps. In the final stage, attention should be given to extending and maintaining of behaviour change [4].

A lower PAM level could possibly lead to poorer health outcomes, as studies have shown that a lower PAM level is associated with poorer HbA1c control, fewer feet checks and eye examinations, lower rates of regular physical exercise, and more use of hospital resources [4–6].

Studies concerning differences in patient activation between men and women with chronic diseases show contradictory results [6–10]. Two studies found a higher level of patient activation in men [7, 9], whereas three other studies did not find a difference between men and women in the level of patient activation [6, 8, 10]. However, all of these studies did not adjust for some important factors, which could have influenced the relation between gender and patient activation. Women with T2D have a lower degree of well-being, a lower health-related quality of life, and a higher body mass index (BMI) and use more often insulin whereas they are less often smokers and have less macrovascular complications compared to men with T2D [11–13]. Well-being, physical health status, and BMI are all associated with patient activation [8]. Therefore, we hypothesized that differences in patient activation between men and women might possibly be influenced by well-being, quality of life, and lifestyle factors. If differences in patient activation between men and women exist, this may indicate that the level of self-management tasks should be more gender specific to achieve optimal health outcomes in both genders. It is unknown whether there are other factors associated with patient activation in men or women. Identifying these associations may indicate gender specific factors to focus on when improving patient activation. Therefore, the aim of our study was to investigate whether the level of patient activation differs between men and women with T2D. Furthermore, we have investigated whether there are other factors associated with degree of patient activation in men compared to women.

## 2. Materials and Methods

### 2.1. Study Population and Setting

**Figure 1:**
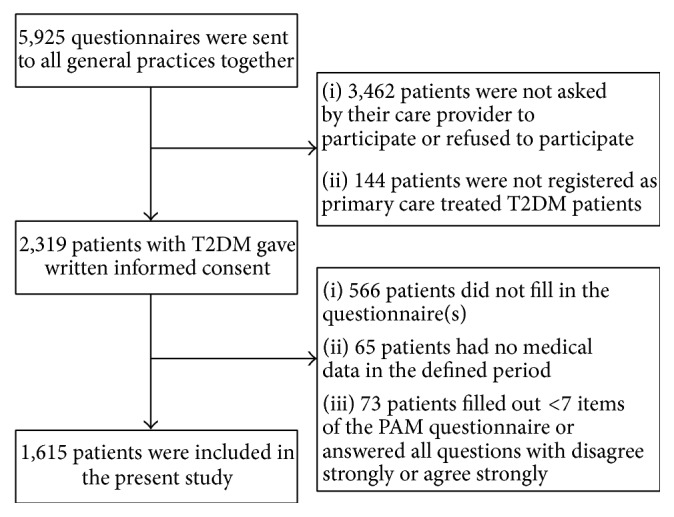
Flowchart of inclusion.

The study population consisted of patients with T2D who were treated in primary care in three regions in the eastern part of the Netherlands. These patients were approached for a quality assessment concerning patient satisfaction performed by Medrie, an organization which facilitates and supports general practitioners (GPs). All patients were asked by their care provider to fill out a survey including questionnaires on quality of life, level of patient activation, and experience with the received care. Patients were included in the period from July 2014 until April 2015. A total of 5925 sets of questionnaires were sent to all general practices in the regions together. All general practices were asked to invite patients with T2D to fill out these questionnaires. Finally, 2319 patients with T2DM gave written informed consent. The other patients refused participation or the GPs did not include the requested number of patients. The final study sample consisted of 1688 (72.8%) patients; see for more details the flowchart in Figure 1.

### 2.2. Patient Activation Questionnaire

In this study the Dutch version of the PAM was used which was validated by NIVEL (Netherlands Institute for Health Services Research) [9]. The questionnaire consists of 13 items which measure knowledge, skills, confidence, and behaviours needed for self-management. Each item has five different response categories: (1) disagree strongly, (2) disagree, (3) agree, (4) agree strongly, and (5) not applicable. In the current study, the same scoring rules as in the Dutch validation study of the PAM were used [9]. Patients who filled out less than 7 items or who answered all items with disagree strongly or agree strongly were excluded. Subsequently, mean scores for the PAM were calculated leaving out items which were responded to with not applicable. The mean scores were transformed into a PAM score ranging from 0 to 100 based on scoring rules of Insignia Health [14]. Based on the same rules, the PAM score was also converted into the four levels of patient activation.

### 2.3. Data Collection

All patients filled out a survey which consisted of the PAM questionnaire for measuring degree of patient activation, the WHO-5 for measuring well-being, and the EQ5D for measuring quality of life [15–17]. The WHO-5 questionnaire consists of descriptions of five different positive feelings: “I have felt cheerful and in good spirits,” “I have felt calm and relaxed,” “I have felt active and vigorous,” “I woke up feeling fresh and rested,” and “my daily life has been filled with things that interest me.” Each feeling has 6 answer options ranging from 0 (not present) to 5 (constantly present) [15]. The EQ5D measures health-related quality of life on five health dimensions: mobility, self-care, usual activities, pain/discomfort, and anxiety/depression. Each dimension has 3 answer options: no problems, some problems, and extreme problems [16]. In this study, the sum scores for the WHO-5 and EQ5D were used.

Demographic and clinical data were collected from the personal health record systems of the GPs. These data were collected during the annual check-up of the patients by their GP and were already routinely sent to the Diabetes Centre (Zwolle, the Netherlands) for benchmark and study purposes. Clinical data that were collected in the period from 9 months before till 5 months after the questionnaire were used. The following data were extracted: age, gender, diabetes duration, BMI, smoking status, HbA1c, use of glucose lowering medication, and the presence of micro- and/or macrovascular complications. The presence of microvascular complications was defined as having microalbuminuria, diabetes retinopathy, and/or diminished sensibility of the feet. The presence of macrovascular complications was defined as (a history of) angina pectoris, myocardial infarction, percutaneous transluminal coronary angioplasty, coronary artery bypass grafting, stroke or transient ischemic attack, or the use of thrombocyte aggregation inhibitors.

### 2.4. Statistical Analysis

Statistical analyses were performed using SPSS version 22 (IBM Corporation, Somers, NY, USA). Multiple imputations were used for missing data on the independent variables, assuming that data was missing at random (MAR) or completely at random (MCAR). Ten imputed datasets were created and the pooled results are described. The patient characteristics are expressed as mean with standard deviation (SD) or median with interquartile range (IQR) for normally distributed and nonnormally distributed data, respectively. Categorical variables are described in numbers and percentages. Differences were considered to be significant at a *p* value of <0.05. The association between gender and patient activation was investigated with multivariate linear regression using the continuous PAM score. Four models were used: (1) a crude model, (2) a model adjusted for age, (3) a model adjusted for age, well-being, quality of life, BMI, smoking, presence of macrovascular complications (MVC), and the use of insulin, and (4) an explorative model with all variables in model (3) and the following diabetes-related factors: HbA1c, diabetes duration, use of oral glucose lowering drugs, and the presence of microvascular complications. These diabetes-related confounders were added to investigate whether the burden of T2D may confound the relation between gender and PAM. Interaction was tested at the 0.10 probability level between gender and well-being, gender and quality of life, gender and BMI, and gender and smoking in models (3) and (4). Interaction terms were only tested if interaction was plausible based on theoretical grounds, and they were only included in the fully adjusted model when they were statistically significant. Stratified analyses according to gender were performed to investigate which factors are associated with patient activation in men and women. For this purpose, model (3) and model (4) were used. The degree to which the different models determined the PAM score was evaluated by the explained variance, shown as adjusted *R* ^2^. Before analyses, the WHO-5 and EQ5D scores were tested for presence of multicollinearity.

### 2.5. Ethical Approval

All patients gave written informed consent for the use of the survey data and the clinical data. According to Dutch guidelines this research does not fall under the scope of the Medical Research Involving Human Subjects Act, and therefore this study does not need a formal approval of an accredited METC (The Medical Ethics Committee of the Isala, Zwolle, the Netherlands).

## 3. Results

### 3.1. Patient Characteristics

The patient characteristics are described in Table 1. Fifty-four percent of the patients were male. Mean age was 67.1 (SD: 9.2) years in men and 68.9 (SD: 10.1) years in women, who were significantly older than men (*p* < 0.001). Men had significantly higher scores on the WHO-5 and EQ5D questionnaires compared to women. Men smoked more frequently and they had also more often micro- and macrovascular complications compared to women. The BMI was significantly higher in women. A higher percentage of men used oral glucose lowering drugs.

### 3.2. Association of Gender

The median PAM score and the distribution of the PAM levels are described in Table 1. The median PAM score was 55.6 (IQR: 51.0–63.1) in men and 55.6 (IQR: 48.9–61.9) in women. The distribution of the PAM levels did not significantly differ between men and women (*p* = 0.294). The results of the regression analyses are described in Table 2. In all models gender was not associated with the PAM score. In the final model (model (3)) a lower age (*b* = −0.13; *p* < 0.001), a higher WHO-5 score (*b* = 0.16; *p* < 0.001), and a lower BMI (*b* = −0.16; *p* < 0.010) were associated with a higher PAM score. No interaction was found between gender and WHO-5 score, gender and EQ5D score, gender and BMI, and gender and smoking. In the explorative model (model (4)), all diabetes-related factors (HbA1c, diabetes duration, use of oral glucose lowering drugs, and the presence of microvascular complications) were not associated with the PAM score. Adding these diabetes-related variables did not significantly affect the results of model (3).

### 3.3. Stratified Analyses According to Gender

Stratified analyses according to gender are described in Table 3. In men, lower age (*b* = −0.18; *p* = 0.001), a higher WHO-5 score (*b* = 0.15; *p* < 0.001), and a lower BMI (*b* = −0.220; *p* = 0.013) were associated with a higher PAM score in model (3) (*R* ^2^ 8.5%). In women, a higher WHO-5 score (*b* = 0.17; *p* < 0.001) and the absence of macrovascular complications (*b* = −2.35; *p* < 0.031) are associated with a higher PAM score in model (3) (*R* ^2^ 10.7%). In the explorative model (model (4)), no associations were found between HbA1c, diabetes duration, use of oral glucose lowering drugs, the presence of microvascular complications, and the PAM score in men or women.

## 4. Discussion

The results of this study show that no difference in degree of patient activation was found between men and women with T2D treated in primary care. Within men, age, well-being, and BMI were found to be associated with degree of patient activation whereas, in women, well-being and MVC were related to patient activation.

Although some studies have investigated differences in patient activation between men and women [6–10], our study is the first study having investigated this relationship in particular. Two other Dutch studies among patients with chronic diseases found a slightly higher level of patient activation in men [7, 9]. Another Dutch study among patients with chronic diseases could not indicate gender as an explanatory determinant for patient activation [8]. Furthermore, two studies from the USA in patients with T2D could also not ascertain a relation between gender and patient activation [6, 10]. However, all of these studies did not adjust for gender-related confounders. Women with T2D have a lower degree of well-being, a lower health-related quality of life, and a higher BMI compared to men with T2D [11, 12]. Well-being, physical health status, and BMI are all associated with patient activation [8]. Therefore, these factors may confound the effect of gender on patient activation. Although these factors differ between men and women in our present study, adjusting for these factors did not influence the relation between gender and patient activation.

We found that a lower degree of well-being was associated with a lower level of patient activation in both men and women. It attributed for 73% and 90% to the explained variance in the final model in men and women, respectively (data not shown). Although a strong association between well-being and patient activation seems to be present, the effect of well-being is rather small as the total explained variance was only 8.4% and 10.4% in the final models for men and women, respectively. A low degree of well-being could indicate the presence of depression, which was found to be associated with patient activation in a previous Dutch study among patients with chronic diseases [8, 18]. This association is not surprising as one can imagine that the inability to feel pleasure (anhedonia), which is one of the main symptoms of depression, will lead to a low level of patient activation [19]. On the other hand, patients with low patient activation are less capable of performing adequate self-management tasks which may lead to lower well-being. The direction of this association should be investigated in further research. The relationships in men between a lower age and a lower BMI with a higher level of patient activation are in line with the results of previous studies [7, 8]. The relation between macrovascular complications and patient activation, which was found in women in the present study, has not been investigated before. Whether this association is actual gender specific or more a matter of coincidence should be investigated in further research.

Some limitations need to be mentioned. Due to the cross-sectional design, causal conclusions could not be drawn. Furthermore, although we have investigated important confounders, still some potentially important factors were not taken into account. We were not able to adjust for educational level, socioeconomic status, and marital status. Inclusion of those variables might increase the explained variance. Educational status and financial distress, which could be used as markers for socioeconomic status, were associated with patient activation in a previous Dutch study [8]. In the same study, living together versus alone was not associated with patient activation. It should be investigated further whether adjusting for these factors will lead to a difference in patient activation between men and women. At last, selection bias could have occurred. However, no difference was found in age or degree of glycaemic control between the study population and the whole T2D population from the three regions.

As no differences between patient activation level between men and women exist, there is no indication that the approach to men and women with respect to self-management tasks should be different. However, this does not directly mean that the same self-care tasks could be given to men and women, as the effectiveness of self-care interventions could still be different. This should be investigated in further research.

## 5. Conclusion

There is no difference in the degree of patient activation of men and women with T2D. Furthermore, no significant influence was found for well-being, quality of life, BMI, and smoking on the relationship between gender and patient activation. Age, well-being, and BMI were found to be associated with patient activation in men, whereas well-being and macrovascular complications were found to be associated with patient activation in women. Based on these results, there is no indication that different levels of self-management tasks should be given to men and women with T2D.

## Figures and Tables

**Table 1:** Patient characteristics.

	Men	Women	*p* value
*n* (%)	874 (54.1)	741 (45.9)	—
Mean age (years)	67.1 (9.2)	68.9 (10.1)	<0.001
Median WHO-5 score	76 (60–80)	72 (52–80)	<0.001
Median EQ5D score	0.9 (0.8–1.0)	0.8 (0.8–1.0)	<0.001
Median BMI	28.0 (26.0–31.4)	30.0 (26.7–34.0)	<0.001
Smoking, *n* (%)	136 (15.6)	73 (9.9)	0.001
MVC, *n* (%)	411 (47.0)	244 (32.9)	<0.001
Use of glucose lowering drugs, *n* (%)	697 (79.7)	560 (75.6)	0.047
Use of insulin, *n* (%)	144 (16.5)	133 (17.9)	0.466
Median diabetes duration	8.3 (4.8–12.1)	8.3 (4.4–12.7)	0.884
Median HbA1c (mmol/mol)	50 (45–56)	51 (45–57)	0.426
Microvascular complications, *n* (%)	401 (45.9)	292 (39.4)	0.006
Median PAM score	55.6 (51.0–63.1)	55.6 (48.9–61.9)	0.235
PAM level			0.294
1	151 (17.3)	131 (17.7)	
2	200 (22.9)	187 (25.2)	
3	433 (49.5)	334 (45.1)	
4	90 (10.3)	89 (12.0)	

Values are depicted as *n* (%), mean (SD), or median (IQR). Continuous data were analysed using independent *t*-tests or the Mann-Whitney *U* test. Categorical variables were analysed using Chi-square tests.

BMI: body mass index; MVC: macrovascular complications.

Number of patients with missing values: WHO-5: 15, EQ5D: 46, BMI: 23, smoking: 25, MVC: 352, diabetes duration: 8, HbA1c: 22, and microvascular complications: 352.

**Table 2:** Multivariate regression analysis for patient activation.

Variables	Model (1)		Model (2)		Model (3)		Model (4) (explorative)	
	Adjusted *R* ^2^ (%) = 0.0%		Adjusted *R* ^2^ (%) = 0.1%		Adjusted *R* ^2^ (%) = 9.6%		Adjusted *R* ^2^ (%) = 9.5%	
	*b* (95% CI)	*p* value	*b* (95% CI)	*p* value	*b* (95% CI)	*p* value	*b* (95% CI)	*p* value
Gender	−0.317 (−1.552, 0.917)	0.614	−0.135 (−1.372, 1.102)	0.830	1.060 (−0.179, 2.300)	0.094	1.062 (−0.189, 2.134)	0.096
Age			−0.102 (−1.660, −0.380)	0.002	−0.130 (−0.197, −0.063)	<0.001	−0.129 (−0.199, −0.058)	<0.001
WHO-5 score					0.158 (0.124, 0.193)	<0.001	0.158 (0.123, 0.192)	<0.001
EQ5D score					3.835 (−0.277, 7.948)	0.068	3.958 (−0.202, 8.118)	0.062
BMI					−0.155 (−0.272, −0.038)	0.010	−0.155 (−0.272, −0.037)	0.010
Smoking					1.473 (−0.327, 3.273)	0.109	1.434 (−0.379, 3.248)	0.121
MVC					−1.386 (−2.813, 0.041)	0.057	−1.436 (−2.879, 0.008)	0.051
Use of insulin					0.158 (−1.408, 1.724)	0.843	0.668 (−1.112, 2.449)	0.462
Diabetes duration							−0.034 (−0.146, 0.078)	0.548
HbA1c							−0.036 (−0.107, 0.034)	0.310
Use of glucose lowering drugs							−0.439 (−1.928, 1.050)	0.563
Microvascular complications							0.206 (−1.467, 1.879)	0.806

BMI: body mass index; MVC: macrovascular complications; *b*: unstandardized regression coefficients.

**Table 3:** Stratified analyses for men and women.

Variables	Men				Women			
	Model (3)		Model (4) (explorative)		Model (3)		Model (4) (explorative)	
	Adjusted *R* ^2^ (%) = 8.5%		Adjusted *R* ^2^ (%) = 8.4%		Adjusted *R* ^2^ (%) = 10.7%		Adjusted *R* ^2^ (%) = 10.4%	
	*b* (95% CI)	*p* value	*b* (95% CI)	*p* value	*b* (95% CI)	*p* value	*b* (95% CI)	*p* value
Age	−0.177 (−0.272, −0.083)	0.001	−0.173 (−0.272, −0.074)	0.001	−0.081 (−0.177, 0.015)	0.097	−0.075 (−0.176, 0.027)	0.150
WHO-5 score	0.152 (0.104, 0.201)	<0.001	0.149 (0.100, 0.198)	<0.001	0.166 (0.116, 0.214)	<0.001	0.166 (0.117, 0.215)	<0.001
EQ5D score	3.828 (−1.947, 9.603)	0.194	3.944 (−1.913, 9.800)	0.187	4.102 (−1.808, 10.013)	0.174	4.371 (−1.616, 10.358)	0.152
BMI	−0.220 (−0.394, −0.047)	0.013	−0.220 (−0.394, −0.046)	0.013	−0.104 (−0.265, 0.057)	0.207	−0.095 (−0.258, 0.068)	0.255
Smoking	1.839 (−0.387, 4.066)	0.105	1.866 (−0.374, 4.106)	0.103	0.894 (−2.153, 3.942)	0.565	0.810 (−2.262, 3.881)	0.605
MVC	−0.594 (−2.371, 1.183)	0.512	−0.704 (−2.500, 1.098)	0.442	−2.346 (−4.473, −0.220)	0.031	−2.333 (−4.468, −0.198)	0.032
Use of insulin	0.451 (−1.708, 2.610)	0.682	1.030 (−1.368, 3.427)	0.400	−0.149 (−2.438, 2.139)	0.898	0.423 (−2.284, 3.130)	0.759
Diabetes duration			−0.049 (−0.205, 0.108)	0.544			−0.024 (−0.185, 0.138)	0.775
HbA1c			−0.028 (−0.121, 0.065)	0.553			−0.055 (−0.164, 0.055)	0.327
Use of glucose lowering drugs			−1.427 (−3.483, 0.629)	0.174			0.750 (−1.452, 2.952)	0.504
Microvascular complications			0.184 (−1.973, 2.341)	0.865			0.115 (−2.358, 2.588)	0.926

BMI: body mass index; MVC: macrovascular complications; *b*: unstandardized regression coefficients.
